# Exploring the molecular reorientations in amorphous rosuvastatin calcium

**DOI:** 10.1039/d0ra06108e

**Published:** 2020-09-11

**Authors:** N. M. Belozerova, P. Bilski, M. Jarek, J. Jenczyk, S. E. Kichanov, D. P. Kozlenko, J. Mielcarek, A. Pajzderska, J. Wąsicki

**Affiliations:** Frank Laboratory of Neutron Physics, Joint Institute for Nuclear Research 141980 Dubna Moscow Region Russia; Faculty of Physics, Adam Mickiewicz University Uniwersytetu Poznańskiego 2 61-614 Poznań Poland jwasicki@amu.edu.pl; NanoBioMedical Centre, Adam Mickiewicz University Wszechnicy Piastowskiej 3 61-614 Poznań Poland; Department of Inorganics and Analytical Chemistry, Poznań University of Medical Sciences Grunwaldzka 6 60-780 Poznań Poland

## Abstract

Molecular reorientations in rosuvastatin calcium, a drug that is widely used to prevent cardiovascular disease, were explored thoroughly by means of solid state nuclear magnetic resonance (^1^H and ^13^C NMR) combined with calculations of steric hindrances. The experimental results reveal rich internal reorientational dynamics. All relaxation processes were tested in a broad range of temperatures and described in terms of their type and the associated energy barriers. The internal molecular mobility of rosuvastatin calcium can be associated with the reorientational dynamics of four methyl groups, accompanied by reorientation of the isopropyl group. The energy barriers of methyl and isopropyl group reorientation depended on the type of *E*/*Z* isomers, while the water content also had a strong influence on the dynamics of the isopropyl group. In the paper, a consistent picture of the molecular dynamics is provided, facilitating our understanding of molecular mobility in this important pharmaceutical solid.

## Abstract 2

Molecular reorientations in rosuvastatin calcium, a drug that is widely used to prevent cardiovascular disease, were explored thoroughly by means of solid state nuclear magnetic resonance (^1^H, ^13^C NMR) combined with calculations of steric hindrances.

## Graphical abstract

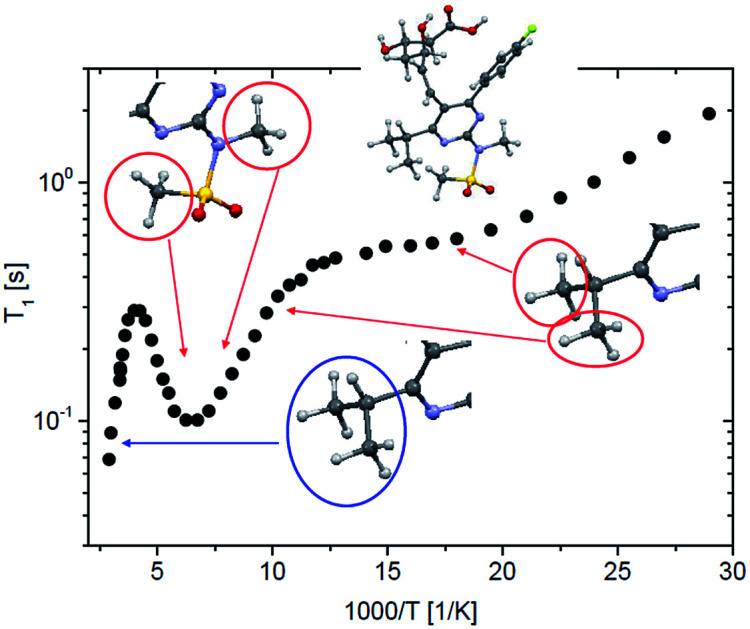

## I. Introduction

A great number of active pharmaceutical ingredients (API) show low bioavailability due to their poor solubility. However, their amorphous form is usually characterised by higher solubility. On the other hand, the use of amorphous substances is associated with problems due to their low physical stability.^1–4^ It is generally believed that knowledge of molecular dynamics, which is determined by intra- and intermolecular interactions, is of great importance in understanding the physicochemical properties of amorphous systems and for development of methods for enhancement of their physicochemical stability.^5,6^ In particular it has been found that the rate of global mobility (α-relaxation) close to the glass transition correlates with the tendency to crystallisation of APIs.^7–9^ Moreover, the molecular reorientations described by the so-called local mobility (β-relaxation)^7,9,10^ can influence on the recrystallization of amorphous systems. The information on molecular mobility is obtained mainly from the measurements by broadband dielectric spectroscopy (BDS). Molecular reorientations in amorphous systems are also studied by nuclear magnetic resonance and quasielastic neutron scattering methods.^5,11–18^ On the other hand, attempts of the identification of molecular reorientations on the basis of analysis of the potential energy as a function of the angle of rotation of a molecular fragment, the so-called potential energy landscape^8^ are much less frequently.

Statins were first introduced into clinical practice in the 1980s.^19^ Depending on their origin, they have a different molecular structure. In the active form the common fragment of the structure is the β-dihydroxyheptanoic acid chain, which acts as a pharmacophore. The structure of the statin molecule is similar to that found in 3-hydroxy-3-methylglutaryl coenzyme A (HMG-CoA), which catalyzes a key step in the cholesterol biosynthesis pathway.^20^ The mechanism of action of statins, responsible for their main therapeutic effect, is the selective competitive inhibition of HMG-CoA reductase, *i.e.* competition between the substrate and the inhibitor.^21^ Modifications in the structure of statins of natural origin, consisting mainly of replacing the decalin ring with an aromatic ring, led to the synthesis of second-generation statins, which include rosuvastatin (RV).^22^ RV molecule contains isopropyl, fluorophenyl and the 6-membered pyrimidine ring. RV, like other statins, is an example of a drug substance that exists in an amorphous state and several crystalline forms, differing in physical properties and pharmacological activity.^23,24^ The amorphous form of rosuvastatin, which is found in Crestor© (rosuvastatin calcium, RVCa), was approved in 2003 by the Food and Drug Administration (FDA), obtaining the status of superstatins.^25^

**Fig. 1:**
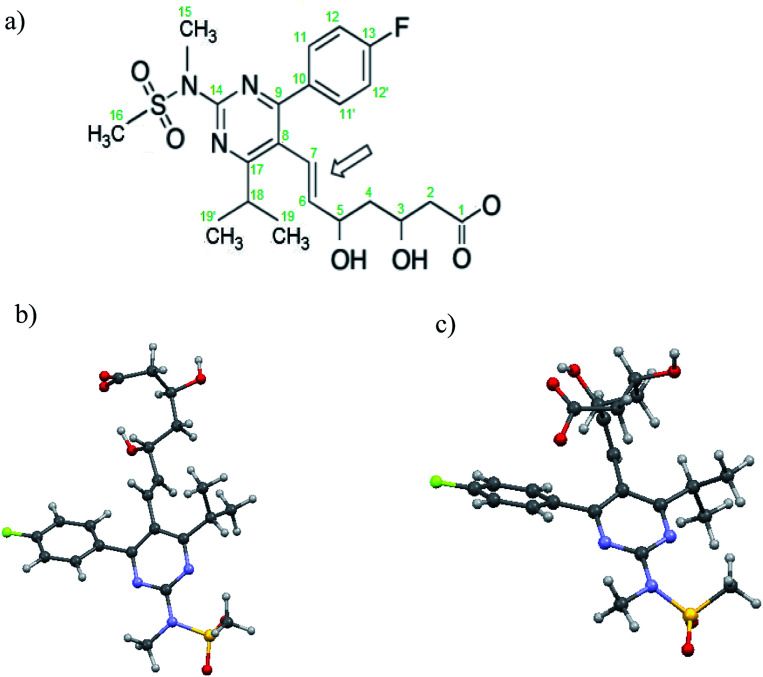
(a) The scheme of the anion of RV along with the numbering of atoms used in this work, the shape of the *E* isomer (b) and *Z* isomer (c) prepared on the basis of data from ref. 28. The arrow indicates the double bond which enables the formation of two isomers.

All statins currently on the pharmaceutical market contain a C=C bond between the heterocyclic core and the chiral dihydroxycarboxy chain, which conditions the existence of *E*/*Z* geometrical isomers. Some statins, including RV, are used in medicine in the form of *E* isomers.^26,27^ On the other hand, there is very little information in the literature on *Z* isomers and one example is the result of studies carried out by Fabris *et al.*^28^ These authors studied RVCa solutions by heteronuclear nuclear magnetic resonance (NMR) as a function of temperature. They showed that for *Z* isomers, unlike *E* isomers, there are conformational motions of large fragments of the RV anion, which are manifested by a significant widening of resonance lines in the ^1^H NMR spectrum (at room temperature).^28^ It was found that the conformational motion observed experimentally is associated with the rotation of the aliphatic chain fragment around a single C_5_–C_6_ bond and the heterocyclic moiety around the C_7_–C_6_ bond (see Fig. 1a). Conformational equilibria and intrinsic preferences of *Z*-isomeric rosuvastatin analogs provide valuable insight into conformational variability, which is important for studying potential interactions within the binding site of the enzyme.

In this paper, we report our attempt to identify and describe molecular reorientation in amorphous RVCa, as well as to determine whether and how the water content of the sample influences anion dynamics. In particular, we plan to check if energy barriers of reorientational motions depend on the type of *E*/*Z* isomers. We combine high-resolution solid-state NMR, temperature dependence on the shape of the NMR line, and relaxation time with a calculation of steric hindrance to understand the internal mobility of RVCa. Understanding the methyl and conformational mobility is an important step in studying the binding site of the enzyme.

## II. Materials and methods

### II.1. The sample

A sample of rosuvastatin calcium (monocalcium bis (+) 7-[4-(4-fluorophenyl)-6-isopropyl-2-(*N*-methyl-*N*-methylsulfonylaminopyrimidin)-5-yl]-(3*R*,5*S*)-dihydroxy-(*E*)-6-heptenoate) as light yellow powder was obtained from Biofarm Sp. z o. o. (Poland) and studied without further purification.

### II.2. Calorimetric measurements

TGA measurements were performed by a TGA4000 thermogravimetric analyser (Perkin Elmer) in a temperature range 300 K to 1173 with a standard rate of 10 K min^−1^ under a dry nitrogen atmosphere (flow rate 20 mL min^−1^).

DSC analyses were performed by a “Perkin Elmer DSC8500” calorimeter. *n*-Dodecane and indium standards were used for instrument calibration. Heating thermograms were carried out at a standard rate of 10 °C min^−1^ under a dry nitrogen atmosphere (flow rate 20 mL min^−1^).

### II.3. Powder X-ray diffraction

The powder X-ray diffraction (PXRD) studies were carried out on powdered samples using an Empyrean (PANalytical) diffractometer with Cu Kα radiation (*λ* = 1.54 Å), reflection-transmission spinner (sample stage) and a PIXcel 3D detector, operating in Bragg–Brentano geometry. Scans were recorded at room temperature (300 K) in angles ranging from 5 to 70 (°2Theta) with a step size of 0.006 and continuous scan mode.

### II.4. ^13^C and ^1^H high-resolution NMR

^1^H spectrum for rosuvastatin dissolved in deuterated water was recorded on a 800 MHz Agilent spectrometer using a HCN triple resonance probe. High-resolution solid-state ^13^C and ^1^H NMR spectra were acquired at ambient conditions on a 400 MHz Agilent spectrometer equipped with Wide Bore Triple Resonance T3 MAS XY probe. Samples were placed into a 4 mm diameter zirconia rotor. Spin–lattice relaxation times *T*_1_ for protons were estimated using a Cross-Polarization (CP) Magic Angle Spinning (MAS) inversion recovery sequence. CP contact time was set 2300 μs and ^13^C detection with dipolar decoupling of protons was used. Spin–lattice relaxation times *T*_1_ for ^13^C were estimated using a simple two-pulse sequence for observing magnetization recovery with the repetition time set to 40 s. All experiments were conducted at ambient temperature, and under magic angle spinning conditions with a spinning frequency of 8 kHz. The ^13^C chemical shifts were referenced to the 38.3 ppm signal of adamantane.

### II.5. ^1^H NMR

The powder samples for ^1^H NMR studies were sealed off in glass ampoules. The measurements of the second moment of the proton NMR line were performed on a continuous wave spectrometer operating on protons at a frequency of 28 MHz (El-Lab Tel-atomic) in a temperature range from 300–80 K. The proton spin–lattice relaxation time *T*_1_ was measured on a pulse spectrometer working at 25 MHz (El-Lab Tel-atomic) by using the saturation recovery method as a function of temperature. The relaxation time in the rorating frame (*T*_1ρ_) was measure with the magnetic field *B*_1_ = 18 G. The temperature of the sample was controlled by means of a gas-flow cryostat and monitored with a Pt resistor to an accuracy of 0.1 K.

### II.6. Calculations

Calculations of steric hindrances for the isolated RV anion were performed using the atom–atom potential method.^29,30^ The interaction energy between two unbounded atoms is described by the formula:1
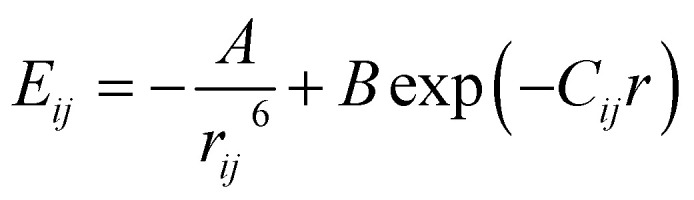
where *r*_*ij*_ is the distance between atoms *i* and *j*; *A*, *B*, *C* are constants characteristic for given atoms. The potential energy for the molecule/ion is the sum of all pairs of interacting atoms:2
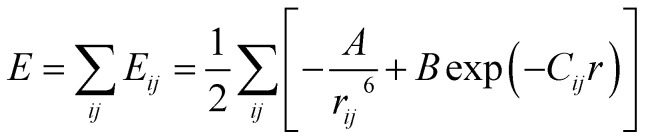


The values of constants *A*, *B*, *C* from ref. 31 were used for the calculations. Constants *A* and *B* between different types of atoms were calculated according to the geometric rule, whereas constant *C* was calculated according to the arithmetic rule. This set of constants has already been used successfully in comparable cases, such as for diazepam,^32^ sibutramine hydrochloride,^33^ chlorpropamide^34^ or to predict of organic crystal structures.^35,36^

The second moment *M*_2_ of ^1^H NMR line was calculated using the Monte Carlo method, as described in detail in ref. 37. The *M*_2_ can be defined as the square of the average local magnetic field *B*, induced by dipole–dipole interaction at the position of resonant nuclei by all nuclei endowed with dipolar magnetic moment, and for the resonance nuclei of the same type it can be expressed as:^38,39^3
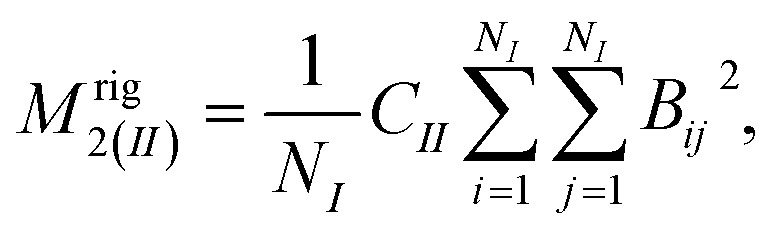
where 
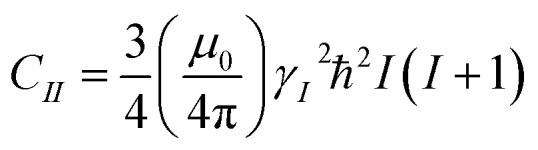
, 
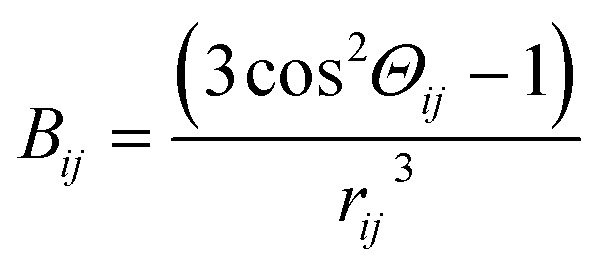
, *N* – number of spins, *I* – the resonant spins, *Θ* – the angles between the spin–spin vector and the direction of the external magnetic field, *r*_*ij*_ – the spin–spin distances, *μ*_0_ – the vacuum permeability, *γ*_*i*_ – gyromagnetic ratio of resonant nuclei. Using formula (3) it is possible to calculate the so-called rigid value of the second moment, when all possible motions take place at frequencies much lower than that corresponding to the width of the absorption line for the absolutely rigid structure. With increasing temperature the *M*_2_ value decreases due to the averaging of the dipolar interactions caused by the motions of the nuclei. In order to calculate the averaged second moment value the *B*_*ij*_ term which depends on relative orientation of interacting spins with respect to the direction of the external magnetic field, as well as on the distance between the interacting spins, should be averaged. It can be performed analytically only for a few simple cases, whereas for more complex motions it is necessary to use numerical methods, such as Monte Carlo.^37,40^

## III. Results and discussion

### III.1. Samples characterisation and their preparation

Fig. 1 shows the schema of the anion of RV and the shape of this anion in *Z* and *E* geometry. In Fig. 1a the arrow indicates the double bond (C_6_=C_7_), which enables the formation of two isomers.

**Fig. 2:**
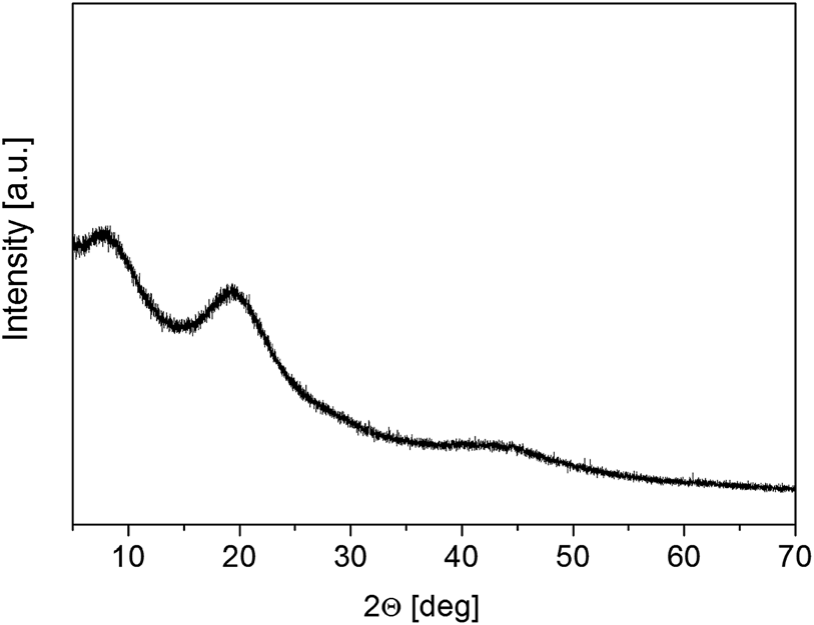
PXRD spectrum for the RVCa sample (1).

The wide-angle X-ray powder diffraction spectrum (PXRD) was recorded for the RVCa sample at room temperature (Fig. 2). No crystal Bragg diffraction peaks were observed in the spectrum, only three broad maxima for 7, 20 and 45 deg. The PXRD spectrum confirms that RVCa is in an amorphous form.

The next stage of the RVCa sample characteristics were differential scanning calorimetry (DSC) measurements, which were carried out in the following cycles: (i) cooling from room temperature to 180 K, (ii) heating from 180 K to 400 K, (iii) cooling from 400 K to 180 K, (iv) heating from 180 K to 400 K.

**Fig. 3:**
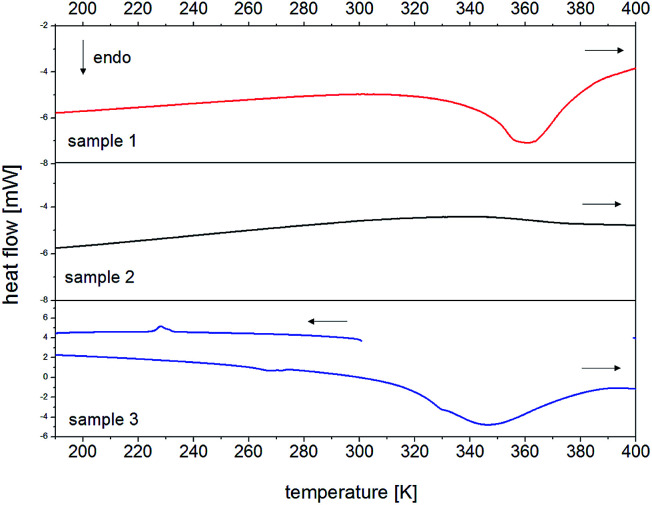
DSC curves for RVCa during heating, for sample 1 (top), sample 2 (middle) and sample (3) bottom. Detailed description in the text.

Analysis of DSC curves at the first heating of the sample (cycle ii) revealed the endothermic peak with a maximum for a temperature of about 360 K, which should be attributed to the process of dehydratation. This interpretation is confirmed by the fact that during the second heating (cycle iv), this effect was no longer observed. Therefore, DSC measurements showed that the RVCa sample was hydrated and the water content in this sample determined on the basis of termogravimetry (TGA) measurements is equal to two water molecules per RVCa unit. This sample is denoted hereafter as sample (1). The anhydrous RVCa sample obtained by heating for 4 hours at 343 K (this process is described in detail in section III.5) was denoted as sample (2). The sample (3) with eight water molecules per RVCa unit was obtained after storing the sample (1) in an environment with 100% humidity for 7 days. As before, the water content was determined on the basis of TGA measurements. The DSC measurements for these samples (2 and 3) were repeated in four cycles and the DSC curves are presented in Fig. 3. Again, for the sample (3) the endothermic broad peak of about 350 K related with the process of dehydratation was observed. Additionally, the anomalies were recorded at 231 K (during cooling) and 263 K (during heating). For all samples, no anomalies were observed in the repeated cooling–heating cycle. Powder diffraction measurements were also carried out for samples (2) and (3), which showed that these samples are also in an amorphous form. Here, it should be noted that PXRD spectra were once again recorded for all samples studied after 3 years of storage, proving that samples are stable in an amorphous form.

### III.2. ^1^H high-resolution NMR (in solution)

In the paper^28^ one can find ^1^H NMR spectra (600 MHz) for two *E* and *Z* isomers dissolved in D_2_O. Comparing these spectra, it can be seen that almost all peaks have the same chemical shift values. The exception is the peak at 7.7 ppm and 7.5 ppm observed only for the *Z* and *E* isomers, respectively. Additionally, the spectra observed for the *Z* isomer were broader. In our spectrum, we observe all peaks characteristic for RVCa and in particular (the coexistence of both) peaks at 7.7 ppm and 7.5 ppm (is detected), which proves that both the isomers are present in the sample being studied. From the ratio of the intensity of these peaks, it can be estimated that the level of the *Z* isomer is about 15%.

### III.3. ^1^H and ^13^C high-resolution NMR (in solid state)

**Fig. 4:**
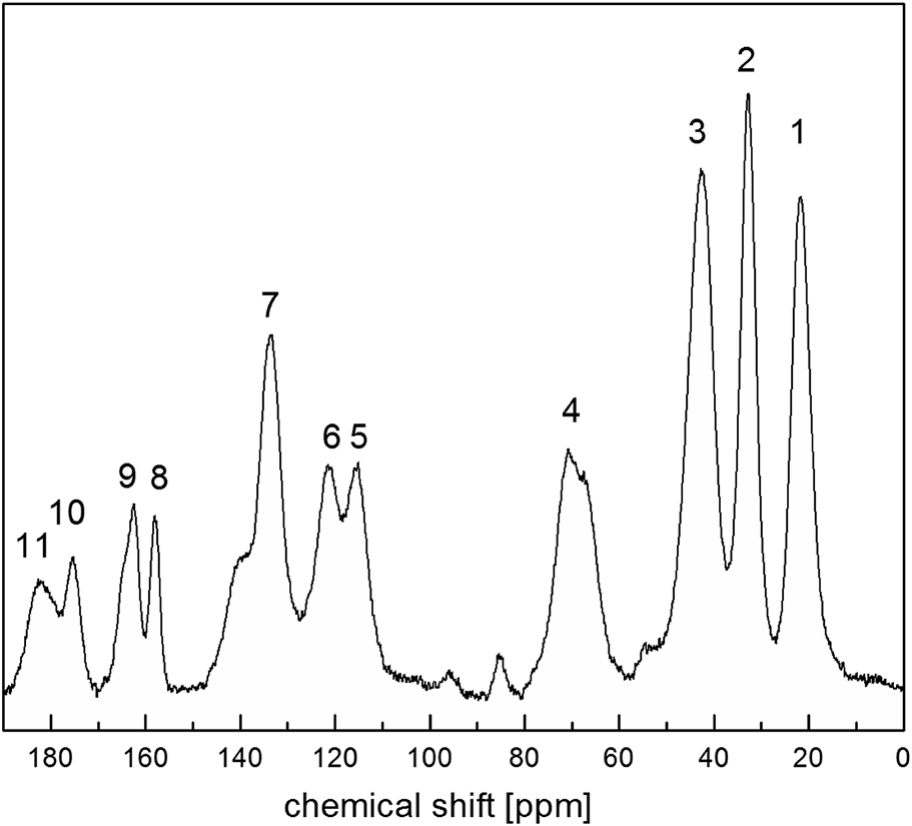
^13^C CP MAS NMR spectrum for the RVCa (sample (1)). The numbers denote the individual peaks.

Fig. 4 shows ^13^C CP MAS NMR spectrum of the RVCa (sample (1)) recorded at room temperature. The spectrum consists of wide lines (numbered from 1 to 11) characteristic for amorphous systems.

Peak assignments of ^13^C CP MAS spectrum were performed by a comparison with ^13^C NMR spectra for RVCa in solutions.^28,41^ The results obtained are summarized in Table 1.

**Table 1:** Chemical shifts of NMR peaks and their assignments, *T*_1_ relaxation time values obtained from ^13^C CP MAS spectra for the RVCa (sample (1)). The accuracy of determining the relaxation times is about 10%

Signal no.	Chemical shift [ppm]	Carbon nucleus	*T*_1_ [s]
1	21.7	C_19_, 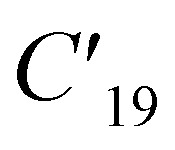	0.58
2	32.8	C_18_, C_16_	9.78
3	42.6	C_15_, C_2_, C_14_	5.98
4	70.7	C_5_, C_3_	15.24
5	115.4	C_12_, 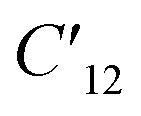	17.78
6	121.3	C_7_, C_8_	
7	133.5	C_11_, 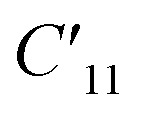 , C_10_, C_6_	10.78
8	157.9	C_14_	16.28
9	162.4	C_13_, C_9_	
10	175.2	C_17_	23.00
11	182.3	C_1_	

The broadening of the NMR signal is due to the fact that the individual carbon atoms in the amorphous sample are in slightly different environments. It should also be noted that some lines are related to several carbon atoms at different positions except for lines number 1, 5, 8, 10 and 11, which are related to individual carbon atoms or equivalent atoms (like 12 and 12′ or 19 and 19′).

**Fig. 5:**
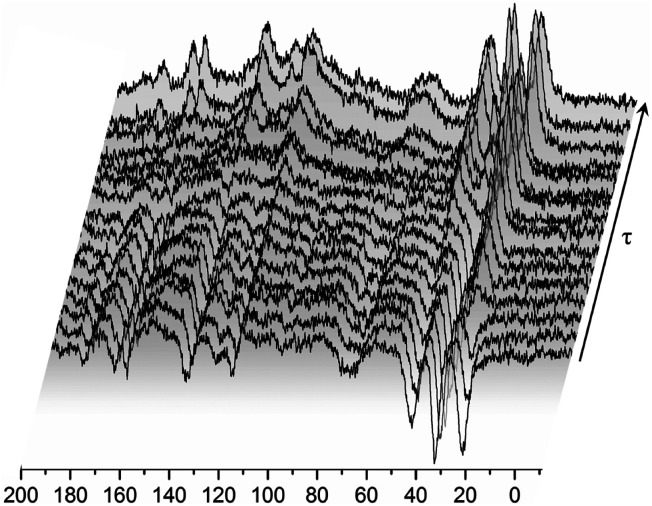
^13^C CP MAS NMR spectra recorded for the RVCa (sample (1)) for different time intervals *τ* between radio-pulses at room temperature.

In order to obtain information on the molecular dynamics of the RV anion, relaxation time measurements were obtained from solid-state high-resolution spectra measured for protons and carbon atoms (^1^H and ^13^C). The spin–lattice relaxation times measured for protons revealed quite a uniform response across the whole molecule *i.e.* the signal recovery is roughly the same for all NMR peaks observed (*T*_1_ ∼ 1 s). It is known, however, that due to spin diffusion, ^1^H may not be a reliable probe to differentiate between the dynamics of the adjacent residue. Therefore, in order to monitor molecular dynamics in depth, it is much better to probe ^13^C relaxation times instead. Although this experiment suffers from low sensitivity (low signal to noise ratio) and substantial time consumption (relatively long spin–lattice relaxation times observed for carbons), it enables the differences between the mobility of neighbouring moieties to be discerned locally. In spite of tentatively estimated relaxation times *T*_1_ for carbons, the data shown in Fig. 5 provide clear evidence that different moieties exhibit various energy barriers for motion. Correspondingly, there is significant relaxation time distribution across the molecular system observed. For all 11 registered lines, *T*_1_ relaxation times were determined for ^13^C atoms, and the values obtained are given in Table 1.

A magnetization recovery experiment allowed us to estimate the spin–lattice relaxation times for each individual carbon. Magnetization recovers exponentially for all lines and can therefore be described by the equation:

4 *M*_z_ = *M*_o_(1 − 2 exp(−*t*/*T*_1_))

where *M*_0_ – is a equilibrium magnetization, *T*_1_ – spin–lattice relaxation time.

The values of *T*_1_ relaxation times are relatively long (except for *T*_1_ for line 1), this means that the sample studied, and more specifically its skeleton (apart from methyl group reorientation), is relatively rigid. This is in line with the analysis of second moment *M*_2_ of ^1^H NMR line and ^1^H relaxation times *T*_1_ (low- and medium-temperature *T*_1_ minimum), which is discussed in the next sections. Additionally, the very short relaxation time for the 19 and 19′ carbon atoms of the isopropyl group (line no. 1) clearly indicates that this group must perform additional reorientations in addition to the reorientation of the methyl groups.

Summing up, measurements of *T*_1_ relaxation time for ^13^C using the CP MAS method: firstly, these confirmed the amorphousness of the RVCa, secondly, they showed that the RV anion skeleton is relatively rigid, and thirdly, they indicated the reorientation of methyl and the isopropyl group. However, the type of motion of the isopropyl group cannot be concluded from the study performed and further studies are necessary.

### III.4. The calculations of steric hindrances

One of the methods allowing molecular reorientations in a molecule/ion or in a crystal to be identified is to calculate steric hindrance based on the atom–atom potential method.^29,30^ The potential energy is calculated as a function of the rotation angle of the molecule/ion or its fragment around a selected axis passing through a single chemical bond.

These calculations were performed for the isolated RV anions on the basis of optimized RVCa structures for both isomers and their different conformers proposed in ref. 28. Selected ion fragments were rotated around a single chemical bond by 1 deg (in the range from −180 deg to 180 deg) and at each position, the potential energy was calculated using formula (2). The results provided a potential energy landscape for a given type of reorientation.

A test of the correctness of the potential energy landscapes calculated was through a comparison of the results obtained for the rotation of the ion fragment around the axis determined by the C_5_–C_6_ bond (for the *Z* isomer) with the results of similar calculations produced using quantum chemistry methods.^28^ Qualitative agreement between the results allowed the use of the atom–atom method to interpret the results of NMR measurements.

**Fig. 6:**
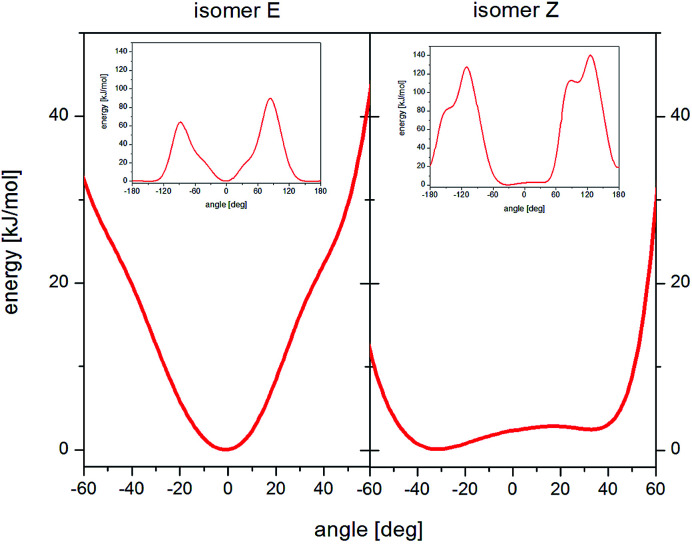
Landscape of energy potential for reorientation of the isopropyl group around the C_17_–C_18_ axis, isomer *E* (left) and isomer *Z* (right).

The analysis of the potential energy landscapes showed small steric hindrance (of a few kJ mol^−1^) which allow reorientation of methyl groups. As expected, the energy curve show 3 minima (distanced by 120 deg), proving the possibility of reorienting the methyl group around the 3-fold axis. The height of the energy barrier (the difference between its maximum and minimum values) of rotation for methyl groups whose carbon atoms are in positions 15 and 16 (Fig. 1a) is similar for the *E* and *Z* isomer (for all three conformers). For the *Z* isomer, similar energy barrier heights were obtained for the methyl groups that are part of the isopropyl group (carbon positions 19 and 19′). However, for the *E* isomer, the height of the energy barrier for reorientation of the methyl groups that are part of the isopropyl group is very different. The methyl group at position 19 has a barrier of about 2 kJ mol^−1^, while the group at position 19′ has about 8 kJ mol^−1^. This is the case for all three *E* isomer conformers. Further analysis of the potential energy landscapes calculated for different RV ion fragments showed that none of them could be rotated by 360 deg, due to the very high energy barrier height. As the results of *T*_1_ relaxation time measurements for ^13^C carbon suggested the motion of the isopropyl group, attention was focused on analyzing the possibility of reorienting this RV anion fragment around the C_17_–C_18_ axis. For the isopropyl group of the *Z* isomer, molecular reorientation is possible through a relatively low energy barrier in the angle range from −40 deg to 40 deg (*i.e.* with an amplitude exceeding 80 deg) (Fig. 6). Interestingly, such a possibility of reorientation was not observed in the calculations for the *E* isomer.

Summarizing, the calculations of potential energy showed that reorientation of all methyl groups are possible for *Z* and *E* isomers. In the *E* isomer, unlike the *Z* isomer, the barrier heights for reorientation of the methyl groups belonging to the isopropyl group differ significantly (almost four times). Reorientations of the isopropyl group with an amplitude of about 80 deg around the C_17_–C_18_ axis are possible only for the *Z* isomer, while for the *E* isomer, the amplitude of reorientations is much smaller.

### III.5. Temperature dependence of the second moment of ^1^H NMR line and relaxation times in the laboratory and rotating frame

In order to determine the height of energy barriers for molecular reorientation identified on the basis of potential energy calculations and ^13^C CP MAS spectra, temperature measurements of ^1^H NMR line shape and relaxation times in the laboratory *T*_1_ and rotating frame *T*_1ρ_ were performed for all three samples (1), (2), (3). Additionally, the aim was to confirm the conclusion on the different isomer content.

**Fig. 7:**
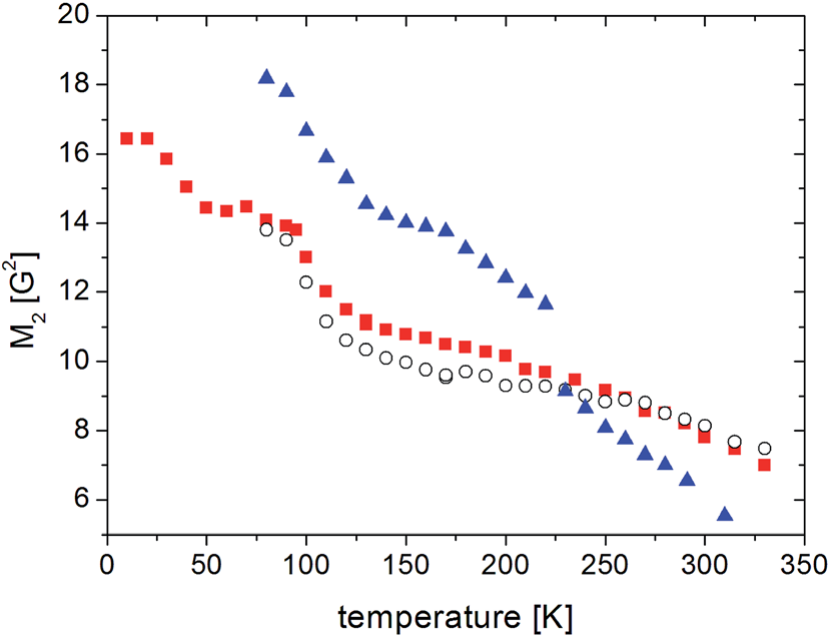
The temperature dependence of *M*_2_ for RVCa samples (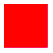) sample 1, (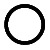) sample 2, (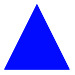) sample 3.

The ^1^H NMR line shape measurements were made in the temperature range from 10 K to 343 K for the sample (1). The temperature dependence of the second moment *M*_2_ of the NMR line is shown in Fig. 7. A constant *M*_2_ value of 16.4 G^2^ was observed for the lowest temperatures (10–20 K), above 20 K the value of *M*_2_ decreased, and in the range from 50 K to 90 K a plateau (of 14.4 G^2^) was detected. Next, in the temperature range from 70 to 130 K *M*_2_ quickly decreases from 14.4 to 11.2 G^2^, while at higher temperatures, 130 K to 343 K *M*_2_ slowly decreases from 11.2 to 7 G^2^.

The reduction observed in the second moment is caused by the averaging of dipole–dipole interactions due to proton motions. Any movement that the molecules or their fragments perform averages the local magnetic field, and if its frequency is greater or comparable to the width of the NMR line, then a reduction in *M*_2_ will be observed.^38,39^

The experimental value of *M*_2_ at the lowest temperature should be treated as a rigid value of the second moment when there is no molecular reorientation (in the NMR time scale) in the system. Knowledge of this value is very important, especially for the analysis of amorphous systems, because it makes it possible to estimate the value of the second moment for intramolecular interactions *M*^inter^_2_, which can be obtained by subtracting the value of intermolecular interactions *M*^intra^_2_ from the experimental value *M*_2_. The *M*^intra^_2_ was calculated based on the structure of a single RVCa anion. The temperature behavior of *M*_2_ can be further explained by calculating averaged *M*_2_ using the Monte Carlo method, as described in detail in ref. 37. The value of the second moment of the NMR line can be calculated by assuming different combinations of methyl group reorientations and different types of molecular reorientations. The comparison of the experimental values of *M*_2_ with the results of the calculations allows molecular reorientations to be identified.

This analysis revealed that the reduction of *M*_2_ in the lowest temperatures should most likely be associated with the reorientation of only one methyl group. The next reduction of *M*_2_ in the temperature range from 70 to 130 K is connected with the reorientation of others methyl groups. The monotonic decrease in *M*_2_ observed for temperatures above 130 K is associated with the oscillation and/or reorientation of a larger fragments of the anion, including reorientation of the isopropyl group.

**Fig. 8:**
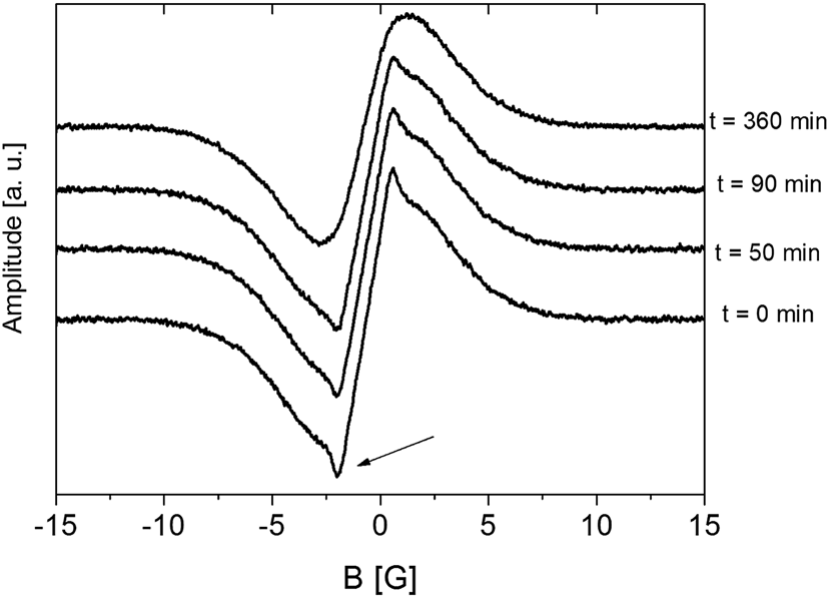
First derivative of the ^1^H NMR line for sample (1) RVCa measured as a function of time at 343 K.

In the higher temperature range (above room temperature), the narrow component of the ^1^H NMR line was observed (Fig. 8, *t* = 0 min). Based on previous research,^33,42^ this narrow line should be attributed to water molecules. As DSC showed water dehydration about 340 K, the ampoule was then opened and ^1^H NMR lines were measured at 343 K as a function of time. It was observed that over time the intensity of the narrow line decreased, and after 4 hours disappeared completely. Therefore, the sample became anhydrous and is denoted as (2). At the end of these measurements, the ampoule was re-sealed and *M*_2_ measurements were done in the temperature range from 343 K to 77 K.

For anhydrous RVCa, the temperature dependence of *M*_2_ is very similar to that of the sample (1). Only in the temperature range from 100 K to 230 K are values of *M*_2_ slightly smaller for the sample (2) compared to the sample (1). For sample (3), *M*_2_ measurements were also performed in the temperature range from 300 K to 80 K. For the sample (3) the second moment increases monotonically with decreasing of temperature, but its values are smaller compared to those for samples (1) and (2) and the reduction of *M*_2_ for sample (3) is more significant compared to samples (1) and (2). At 230 K, the temperature of the anomaly detected on DSC curve, a rapid jump of *M*_2_ is observed. The values of *M*_2_ for the lower temperatures than 230 K are clearly higher compared to other samples.

The lack of difference in the values of *M*_2_ in the lower temperatures for samples (1) and (2) means that the contribution to the second moment coming from rigid (in NMR time scale) water molecules (despite the short distance H⋯H in the water molecule of 1.5 A) is negligible. The reason for this is the negligible number of protons in the water molecules (4 protons/2 anions) relative to the number of protons found in RVCa (54 protons). At higher temperatures, water molecules perform fast reorientations around different axes (molecular tumbling), as evidenced by the narrow component of the ^1^H NMR line that was observed (Fig. 8). In this case, the second moment of water molecules is averaged almost to zero and therefore no difference was observed in the experimental *M*_2_ values for samples (1) and (2). The temperature dependence of *M*_2_ for the sample (1) also indicates that the water molecules are located into the free spaces between the anions, and they do not fundamentally modify the type of molecular reorientation in this sample.

**Fig. 9:**
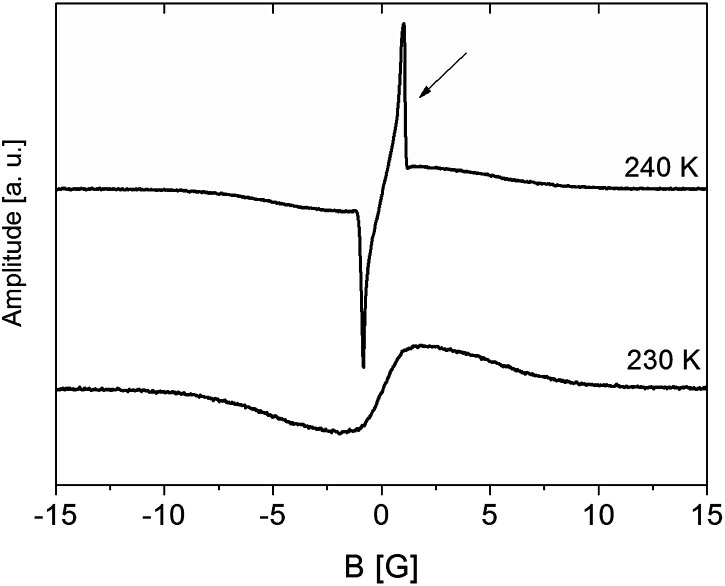
First derivative of the ^1^H NMR line for sample (3) RVCa.

At low and intermediate temperatures (80–200 K), the higher *M*_2_ values for the sample (3) compared to other samples indicate that a significant increase in the number of water molecules results in the formation of its aggregates at these temperatures, which is indicated by a jump of the *M*_2_ value at 230 K and an anomaly on the DSC curve. It is also interesting to compare the shape of the ^1^H NMR line for the sample (3) at 240 K with 230 K (Fig. 9). At a higher temperature, a two-component line was observed, and after cooling the sample by only 10°, the line became clearly single. The narrow component (marked with an arrow) is related to the mobile water molecules, which are rigid (in the NMR scale) at lower temperatures.

The aggregates make a significant contribution to the total second moment value of the sample (3). Because at this temperature range the temperature dependence of *M*_2_ for sample (3) is similar to that observed for samples (1) and (2), in this temperature range, the aggregates formed do not significantly modify the molecular reorientation of the anion. At higher temperatures, the “melting” of aggregates was observed, which results from fast reorientation of water molecules (molecular tumbling) and therefore their contribution to total *M*_2_ is close to zero. The presence of water molecules causes an increase in the volume of the system, which significantly influenced the mobility of anion fragments (including isopropyl group) and which is clearly visible as a more rapid reduction in *M*_2_ above 200 K for sample 3 than for samples (1) and (2).

**Fig. 10:**
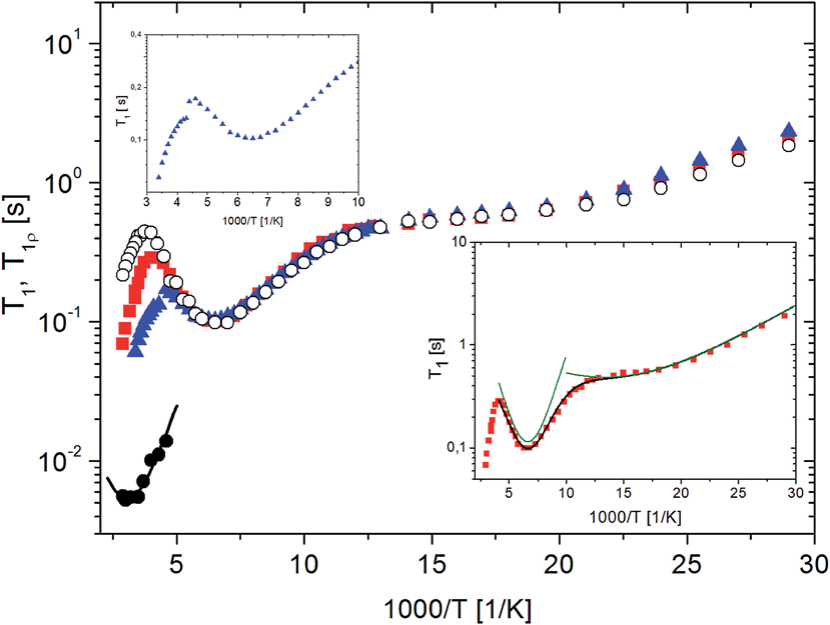
The dependence of proton spin–lattice relaxation time *T*_1_ on the inverse of temperature for RVCa samples (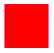) sample 1, (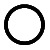) sample 2, (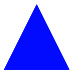) sample 3 and relaxation time in the rotating frame *T*_1ρ_ (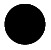) sample 2. The solid line represent the best fit to the experimental point of *T*_1ρ_ (sample 2) using eqn (7). Insert (bottom) – the best fit to the experimental point (for sample 1) using eqn (5) showing low and medium-temperature processes. Insert (top) – the fragment of the dependence of spin–lattice relaxation time *T*_1_ for sample 3.

In order to obtain more precise information for RVCa molecular reorientations, especially for the activation parameters characterizing these reorientations, measurements of proton spin–lattice relaxation times *T*_1_ as a function of temperature were performed for all three samples. These measurements were done in a wide temperature range from 30 K to 348 K, and the results are presented in Fig. 10.

Beginning from the lowest temperatures, the time *T*_1_ shortens as the temperature increases: in the temperature range 50–80 K, the relaxation time shows a weak dependence on temperature and at 154 K, a minimum of 0.1 s is visible. With a further rise in temperature, the relaxation time *T*_1_ increases, reaching the maximum for the sample (1) at 247 K, for sample (2) at 262 K, and for sample (3) at 215 K. With further temperature increases, the relaxation time *T*_1_ is shortened. Therefore, the ln *T*_1_(1000/*T*) dependence shows three clearly separated relaxation processes: (i) low, (ii) medium and (iii) high temperature. However, only the latter process is clearly different for individual samples, while lower and medium processes are related to methyl group reorientation, which do not depend on the sample. For sample (3) the jump of *T*_1_ value at 230 K is also observed.

For the quantitative description of the temperature dependence of time *T*_1_, the classic BPP equation was used:5
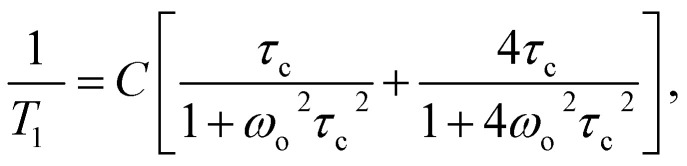

6 where *τ*_c_ = *τ*_0_ exp(*E*_A_/*RT*)

and *C* – relaxation constant, *τ*_0_ – pre-exponential constant, *τ*_c_ – the correlation time, *E*_A_ – the activation energy.

The best fit to the experimental points was obtained when low and medium temperature minima were described by means of two BPP equations. The results obtained are shown in Fig. 10 as solid line, and the activation parameters are summarized in Table 2.

**Table 2:** The activation parameters for reorientation of methyl groups in RVCa (sample (1)) extracted from temperature dependence of *T*_1_

	The relaxation processes	
	Medium	Low
*C* [1/s]	(9.64 ± 0.28) 10^8^	(2.29 ± 0.13) 10^8^
*τ*_o_ [s]	(1.69 ± 0.12) 10^−11^	(58.70 ± 2.44) 10^−11^
*E*_A_ [kJ mol^−1^]	6.84 ± 0.23	1.23 ± 0.08

Analyzing the parameters in Table 2, attention should be paid to the ratio of *C* values for various relaxation processes. These relationships are important for further interpretation of the results, in particular, the ratio of *Z*/*E* isomers.

It should be noted that if the low-temperature process corresponds to the reorientation of one methyl group and the medium-temperature process to the reorientation of three methyl groups, the ratio of the *C* constants from formula (3) for these processes should be 1 : 3 = 0.33. However, the data in Table 2 show that it is 2.29/9.64 = 0.24, which is definitely lower. This means that not every RVCa ion possesses the methyl group, which reorients by the low activation energy barrier. As this low barrier for the reorientation of methyl group (belonging to the isopropyl group) is characteristic of the *E* isomer, it means that the sample does not consist only of the *E* isomer, but is a mixture of *E* and *Z* isomers. Moreover, if the height of the energy barrier for the reorientation of one of the methyl groups from the isopropyl group of the *E* isomer is lower than the height of the barriers for methyl groups belonging to both isomers (which was showed by the calculations in Section III.3), then the ratio of *C* constants for both minima indicates the ratio of isomers in the sample. The percentage of *E* and *Z* isomers thus obtained is 75 and 25, respectively, which is in reasonable agreement with the results obtained from the analysis of the ^1^H spectrum (Section III.1.)

The high temperature process, in contrast to the other two, depends on the water content. As the water content increases, the temperature at which this process can be observed decreases, which indicates that these processes will differ in terms of the height of the energy barrier. The value of *E*_A_ was estimated from the slope of ln *T*_1_(1000/*T*) and is 15.8 and 13.3 kJ mol^−1^ for samples (1) and (3), respectively. Additionally, the measurements were performed for the relaxation time in the rotating *T*_1ρ_ coordinate system. This is the relaxation time in which the sample “feels” the magnetic field *B*_1_ applied as a radio-impulse, which is three orders of magnitude smaller than the external *B*_0_ field. The value of the *B*_1_ field must be greater than the value of the local field generated in the sample by the magnetic moments of the nuclei. In our case, the value of this field was 10 G and measurements were taken in the temperature ranges from 217 K to 344 K. The results for sample (2) are also shown in Fig. 10. The detection of the minimum of *T*_1ρ_ confirms that the high-temperature process of *T*_1_ has an activation character.

The temperature dependence of this minimum is described by the formula:7
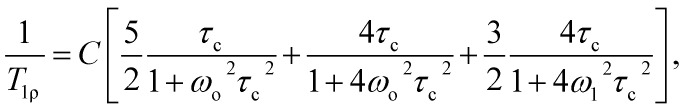
where *ω*_1_ = *γB*_1_, others symbols are the same as in the eqn (5).

In Fig. 10, the best fit to the experimental values obtained using eqn (7) is presented by solid line.

This high-temperature relaxation process should be associated with the reorientation of the isopropyl group, which seems to be confirmed by the calculations of potential energy and ^13^C CP MAS spectra. Interestingly, such reorientations are only possible for the *Z* isomer. Thus, the occurrence of this process confirms the conclusion that the RVCa sample is a mixture of *E* and *Z* isomers.

## IV. Conclusions

^1^H and ^13^C NMR measurements supported by calculations of the potential energy landscape and steric hindrances in the amorphous RVCa sample allowed the molecular reorientation of RV anion to be identified in full. Reorientation of all methyl groups was observed. However, only for the *Z* isomer are energy barriers very similar for all methyl groups and are about 7 kJ mol^−1^, while for the *E* isomer, the energy barrier of reorientation of one methyl group from the isopropyl group is almost four times lower than 8 kJ mol^−1^. Due to this difference, it was possible to estimate the content of the *E* isomer in the RVCa sample at around 80%. This value is close to that determined on the basis of the ^1^H NMR high resolution spectrum in solution (D_2_O).

Another type of motion detected in amorphous RVCa concerns reorientations of the isopropyl group around the axis along the C_17_–C_18_ bond. Such a motion with a significant amplitude of 80° is only possible for the *Z* isomer. This observation provides strong confirmation of the presence of the *Z* isomer in the RVCa sample.

Interesting information on the interactions determining molecular motions was provided by studies on the effects of water content. As the height of the energy reorientation barriers of methyl groups does not depend on water content, this motion is mainly determined by intra-ion interactions. On the other hand, the reorientations of isopropyl groups strongly depend on the degree of water content of the sample, which means that they are determined by both intra- and interionic interactions.

Additionally, powder diffraction spectra recorded after 3 years of storage confirm that all the samples studied are stable in their amorphous form.

## Funding

The work was part-financed by the Plenipotentiary of Poland to JINR, Dubna, Program “A study of the structure and molecular dynamics in crystalline and amorphous therapeutic compounds”.

## Conflicts of interest

The authors declare no conflict of interest.

## Supplementary Material
